# Towards a Standard Psychometric Diagnostic Interview for Subjects at Ultra High Risk of Psychosis: CAARMS versus SIPS

**DOI:** 10.1155/2016/7146341

**Published:** 2016-05-30

**Authors:** P. Fusar-Poli, M. Cappucciati, G. Rutigliano, T. Y. Lee, Q. Beverly, I. Bonoldi, J. Lelli, S. J. Kaar, E. Gago, M. Rocchetti, R. Patel, V. Bhavsar, S. Tognin, S. Badger, M. Calem, K. Lim, J. S. Kwon, J. Perez, P. McGuire

**Affiliations:** ^1^Institute of Psychiatry Psychology and Neuroscience (IoPPN), King's College London, London SE5 8AF, UK; ^2^OASIS Service, South London and Maudsley NHS Foundation Trust, London SE11, UK; ^3^Department of Psychiatry, Seoul National University College of Medicine, Seoul 08826, Republic of Korea; ^4^Cambridgeshire and Peterborough NHS Foundation Trust, Cambridge CB21 5HH, UK; ^5^Real-Time Systems Laboratory, Scuola Superiore Sant'Anna, 56124 Pisa, Italy; ^6^Department of Psychiatry, University of Cambridge, Cambridge CB2 0SZ, UK

## Abstract

*Background*. Several psychometric instruments are available for the diagnostic interview of subjects at ultra high risk (UHR) of psychosis. Their diagnostic comparability is unknown.* Methods*. All referrals to the OASIS (London) or CAMEO (Cambridgeshire) UHR services from May 13 to Dec 14 were interviewed for a UHR state using both the CAARMS 12/2006 and the SIPS 5.0. Percent overall agreement, kappa, the McNemar-Bowker *χ* ^2^ test, equipercentile methods, and residual analyses were used to investigate diagnostic outcomes and symptoms severity or frequency. A conversion algorithm (CONVERT) was validated in an independent UHR sample from the Seoul Youth Clinic (Seoul).* Results*. There was overall substantial CAARMS-versus-SIPS agreement in the identification of UHR subjects (*n* = 212, percent overall agreement = 86%; kappa = 0.781, 95% CI from 0.684 to 0.878; McNemar-Bowker test = 0.069), with the exception of the brief limited intermittent psychotic symptoms (BLIPS) subgroup. Equipercentile-linking table linked symptoms severity and frequency across the CAARMS and SIPS. The conversion algorithm was validated in 93 UHR subjects, showing excellent diagnostic accuracy (CAARMS to SIPS: ROC area 0.929; SIPS to CAARMS: ROC area 0.903).* Conclusions*. This study provides initial comparability data between CAARMS and SIPS and will inform ongoing multicentre studies and clinical guidelines for the UHR psychometric diagnostic interview.

## 1. Introduction

The development of psychometric tools to prospectively identify subjects at ultra high clinical risk (UHR hereafter) of psychosis has allowed preventative screening [1], diagnosis [2], and interventions [3] to be feasible in psychiatry. In 1991, Jackson and McGorry were the first to initiate reliability studies to psychometrically assess first-episode subjects via a semistructured interview in order to ascertain the presence of prodromal symptoms [4]. On the basis of their results, in 1995 Yung and colleagues set up the first clinical service for UHR individuals and conceived the first comprehensive UHR psychometric instrument [5]. The Comprehensive Assessment of At-Risk Mental States (CAARMS hereafter) was developed at the Personal Assessment and Crisis Evaluation (PACE) Clinic in Melbourne [6] and has been widely used in Australia, Asia, and Europe to interview for “At-Risk Mental State, ARMS,” criteria. Their pivotal work resulted in the formulation of three UHR criteria: attenuated psychotic symptoms (APS hereafter), brief limited intermittent psychotic symptoms (BLIPS hereafter), and trait vulnerability plus a marked decline in psychosocial functioning (Genetic Risk and Deterioration syndrome: GRD hereafter). A few years later, in 1999, based on these criteria, Miller et al. (1999) [7] developed a similar psychometric instrument for quantitatively rating symptoms in patients at UHR of psychosis [8], in the Prevention through Risk Identification, Management and Education (PRIME) Clinic in New Haven (USA): the Structured Interview for Psychosis-Risk Syndrome (SIPS hereafter) [8] (for a detailed genealogy of the CAARMS and SIPS see [9, 10]).

The CAARMS and the SIPS address the same construct and use similar criteria, and they can deliver comparable positive predictive values over follow-up time [11, 12]. However, their operationalization differs [10], with substantial changes over different versions of the instruments [10]. Operationalization differences include disparity in psychopathological definitions of the APS, time and frequency criteria, functional decline criterion, BLIPS criteria, assessment of comorbidities, and substance misuse (see Tables 1 and 2 and eTable 1 (in Supplementary Material available online at http://dx.doi.org/10.1155/2016/7146341) for a detailed comparison of CAARMS 12/2006 and SIPS 5.0).

The resulting overall weight of similarities and differences between the two instruments on UHR identification is unknown. Psychometric diagnostic uncertainty questions validity of the UHR diagnostic interview, creating inconsistencies between clinicians or researchers and misunderstandings in patients [13]. Comparability of current clinical, neurobiological, cognitive, and therapeutic UHR research findings may be also questionable and compromised, with the risk of “a profusion of statistically significant, but minimally differentiating" [14] results of limited clinical utility. Psychometric uncertainty may significantly impact the development of future large-scale UHR multicentre studies, by amplifying heterogeneity across individual sites. These concerns and speculations have never been tested empirically. To resolve the “current confusion” [13], research studies allowing “a thorough evaluation of the comparability of samples” [13] have been urgently advocated [10, 15].

We present here the first study addressing the psychometric comparability of the CAARMS 12/2006 [16] versus SIPS 5.0 [8]. Our principal aim was to test if the CAARMS 12/2006 and the SIPS 5.0 can equally identify UHR subjects in a large pool of individuals referred to high-risk services for potential UHR symptoms. Our second aim was to qualitatively investigate potential discrepancies and to link the severity and frequency of symptoms with equipercentile-linking tables. Our third aim was to develop a pragmatic algorithm to convert individual cases across the two instruments, to implement it in an automated conversion package (CONVERT), and to validate it in an independent UHR sample.

## 2. Methods

### 2.1. Samples

We included all referrals to the OASIS (Outreach And Support In South London; http://www.slam.nhs.uk/oasis) and CAMEO (Cambridgeshire and Peterborough Assessing Managing and Enhancing Outcomes, http://www.cameo.nhs.uk/) clinics assessed for a UHR state in the period May 2013–December 2014. The OASIS team was started in 2001 [17] and it is specialized in detecting and treating subjects at UHR of psychosis aged 16–35 [17]. CAMEO was started in 2007 and it is an early intervention in psychosis service which offers management for UHR people aged 17–35 in Cambridgeshire, UK, and provides initial assessments to those under 17. Referrals for both services are accepted from multiple sources including general practitioners, other mental health services, school and college counselors, relatives, and self-referrals [18]. The validation sample for CONVERT included all referrals to the Seoul Youth Clinic assessed for a UHR state with the CAARMS 12/2006 and the SIPS 5.0 as part of the standard clinical practice. The Seoul Youth Clinic was started in 2004 and offers assessment and treatment for UHR people aged 15–35 in Seoul, South Korea [19]. Subjects are recruited from Seoul National University Hospital and other psychiatric clinics and public mental health centers or they can contact the clinic by telephone or an Internet homepage.

### 2.2. Procedure

The study samples were designed to reflect at-risk populations as they are encountered in day-to-day practice: all subjects were drawn from the same pool of people referred to high-risk services because of suspect prodromal signs and symptoms of psychosis. Avoiding the use of external and non-help-seeking control groups who do not reflect the clinical composition of people actually assessed in high risk is essential to properly compare the diagnostic abilities of the two instruments. Furthermore, we only included subjects who were directly assessed with both psychometric instruments during face-to-face interviews, excluding those who declined the full assessment or who were unable to complete it. Responsible clinicians interviewed the participants with the CAARMS 12/2006 [16] and with the SIPS 5.0 [8]. The training procedure and the interrater reliability of the OASIS and CAMEO clinicians have been fully detailed in Supplementary eMethod 1. Subjects accessing the Seoul Youth Clinic are usually assessed with both CAARMS 12/2006 and SIPS 5.0 instruments as part of standard clinical practice, and further details are provided in an independent publication [19].

### 2.3. Clinical Measures

The primary outcome measure was the psychometric diagnosis under the CAARMS 12/2006 [16] versus SIPS 5.0 [8] at the end of the diagnostic interview assessment: at risk of psychosis (UHR+ hereafter), not at risk of psychosis (UHR− hereafter), and Psychosis. Secondary outcome measures included the severity and frequency of UHR as measured on the diagnostic subscales: P1–P4 on the CAARMS 12/2006 and P1–P5 on the SIPS 5.0 (eTable 1). Additional variables recorded were subgroup of UHR+ (APS, BLIPS, and GRD), age, gender, ethnicity, baseline comorbid diagnosis, duration of follow-up, and functional status (GAF in the SIPS 5.0 and SOFAS in the CAARMS 12/2006).

### 2.4. Statistical Analysis

The primary aim of the study was investigated by comparing the diagnostic outcomes (i.e., UHR+, UHR−, and Psychosis) under CAARMS 12/2006 versus SIPS 5.0 in OASIS and CAMEO samples, using IBM SPSS Statistics 22 and STATA 13 software. Specifically, we calculated percent overall agreement, kappa (with its 95% CI), and the exact McNemar-Bowker *χ* ^2^ test to assess marginal errors. Given evidence that baseline functional status is a strong predictor of longitudinal outcome [20], we further performed a weighted kappa analysis, weighting the three groups according to their relative baseline functional level, as established in our previous meta-analysis (i.e., UHR− = 1, UHR+ = 0.84, and Psychosis = 0, eFigure 1, adapted from [20]). We additionally estimated the prevalence and bias adjusted kappa (PABAK) [21] which adjusts the kappa for imbalances caused by differences in prevalence and bias [22]. Interpretation of the kappa values varies, but some guidelines were provided by Landis and Koch (1977) for kappa coefficients suggesting that kappa of 0.01 indicates “poor” agreement; kappa values from 0.01 to 0.20 indicate “slight” agreement; kappa values from 0.21 to 0.40 indicate “fair” agreement; kappa values from 0.41 to 0.60 indicate “moderate” agreement; kappa values from 0.61 to 0.80 indicate “substantial” agreement; kappa values from 0.81 to 1.00 indicate “almost prefect” agreement [23].

The secondary aim of the study was investigated using post hoc explorative residual analyses comparing different subgroups (i.e., UHR−, GRD, APS, BLIPS, and Psychosis), Bonferroni-corrected for multiple comparisons. Qualitative analyses of discrepancies across the two instruments were also conducted, to better elucidate the impact of each specific cell on the overall results. We further converted the severity and frequency of symptoms by employing a linking method, using equate 2.0-3 [24] under R 3.1.2 software. This method is detailed in eMethod 2.

The third aim of the study was investigated with qualitative a priori comparisons of a priori operationalization differences between the CAARMS 12/2006 and SIPS 5.0, as detailed in Tables 1 and 2. Equipercentile linking, percent overall agreement, and kappa estimated above here in the OASIS and CAMEO services were not used for the development of this pragmatic algorithm. A software engineer (JL) then implemented the conversion algorithm in an automated package. CONVERT is a Python application which implements the conversions of individual outcomes (UHR−, UHR+ [GRD, APS, and BLIPS], and Psychosis) between the CAARMS 12/2006 and SIPS 5.0 as proposed by the conversion algorithm. CONVERT takes as input a *∗*.xls file and produces as output a *∗*.xls file. To freely download the tool and the template *∗*.xls input file and get further details please visit https://bitbucket.org/ioppn/convert. We first piloted CONVERT in the OASIS and CAMEO dataset and then validated it in an independent UHR sample recruited at the Seoul Youth Clinic. Diagnostic accuracy measures of CONVERT were analysed with respect to both CAARMS 12/2006-to-SIPS 5.0 and SIPS 5.0-to-CAARMS 12/2006 conversions and included percent overall agreement, kappa (with its 95% CI), PABAK, sensitivity, specificity, and nonparametric Receiver Operating Characteristic (ROC) analyses.

## 3. Results

### 3.1. Samples and Raters Characteristics

The OASIS and CAMEO sample consisted of 212 consecutive help-seeking subjects recruited between May 2013 and December 2014 (OASIS = 128, CAMEO = 84). Of the 212 subjects, 82 were females (38.7%). The mean age was of 24.13 years (SD = 4.98, range = 16–35 years). The majority of subjects were white British (50%), followed by black British (26.1%) and subjects from Asia (4.1%), Africa (4.3%), and South America (1.1%) and from other European countries (11.4%). The mean SOFAS score was of 51.51 (SD = 17.26, median = 48, and range = 18–95). Sociodemographic characteristics of referrals across OASIS and CAMEO are detailed in the Supplementary Material (eTable 2). Each rater interviewed on average 35.33 (SD 31.1) subjects. The IRR for the principal diagnostic outcome (*n* = 21) was of kappa = 0.902 for the CAARMS 12/2006 and kappa = 0.854 for the SIPS 5.0. The IRRs for each subscale were in the excellent range: CAARMS 12/2006 kappa from 0.863 to 0.915 and SIPS 5.0 kappa from 0.815 to 0.923 (eMethod 1).

The validation sample from the Seoul Youth Clinic included 93 UHR subjects, with a mean age of 20.24 years (SD = 4.00, range = 15–33), mostly males (29% females), of Asian ethnicity [19].

### 3.2. Diagnostic Comparison of CAARMS 12/2006 and SIPS 5.0 in OASIS and CAMEO

The percent overall agreement was 86.32% (expected agreement by chance 37.42%) and the kappa was substantial 0.781 (*Z* = 15.83, *p* < 0.001, and 95% CI from 0.684 to 0.878) with the McNemar-Bowker *χ* ^2^ (7.087) resulting in *p* = 0.069. The prevalence and bias adjusted kappa was 0.795 (95% CI from 0.731 to 0.856). The kappa remained substantial when it was estimated in the subset of subjects rated by independent raters (overall agreement 92.86%, expected agreement by chance 76.08%, kappa = 0.701, *Z* = 3.87, and *p* = 0.001). Full details of the main diagnostic comparison are appended in Table 3. When the analysis was weighted for the relative functional impairment of the three groups the results were very similar: the percent overall agreement was 88.43% (expected agreement 45.44%) and the kappa was 0.788 (*Z* = 16.22, *p* < 0.001, and 95% CI from 0.693 to 0.883).

### 3.3. Qualitative Analysis and Equipercentile Linking of CAARMS 12/2006 and SIPS 5.0

Post hoc analyses (see eTable 3) confirmed an overall substantial agreement across the two instruments with the exception of the cell [2,5: CAARMS 12/2006-BLIPS versus SIPS 5.0-Psychosis, adjusted residual = 3.7]. This indicates that, out of the 25 subjects diagnosed with BLIPS by the CAARMS 12/2006, 14 were diagnosed as psychotic by the SIPS 5.0 (eTable 3). The count in this cell was statistically higher than the count expected by chance. To better elucidate these differences we have conducted a qualitative analysis of psychopathological characteristics of these 14 patients, which is appended in eTable 4. The equipercentile linking between severity and frequency scores of the two instruments is detailed in Table 4.

### 3.4. Development and Validation of an Automatized Conversion Algorithm (CONVERT)

**Figure 1:**
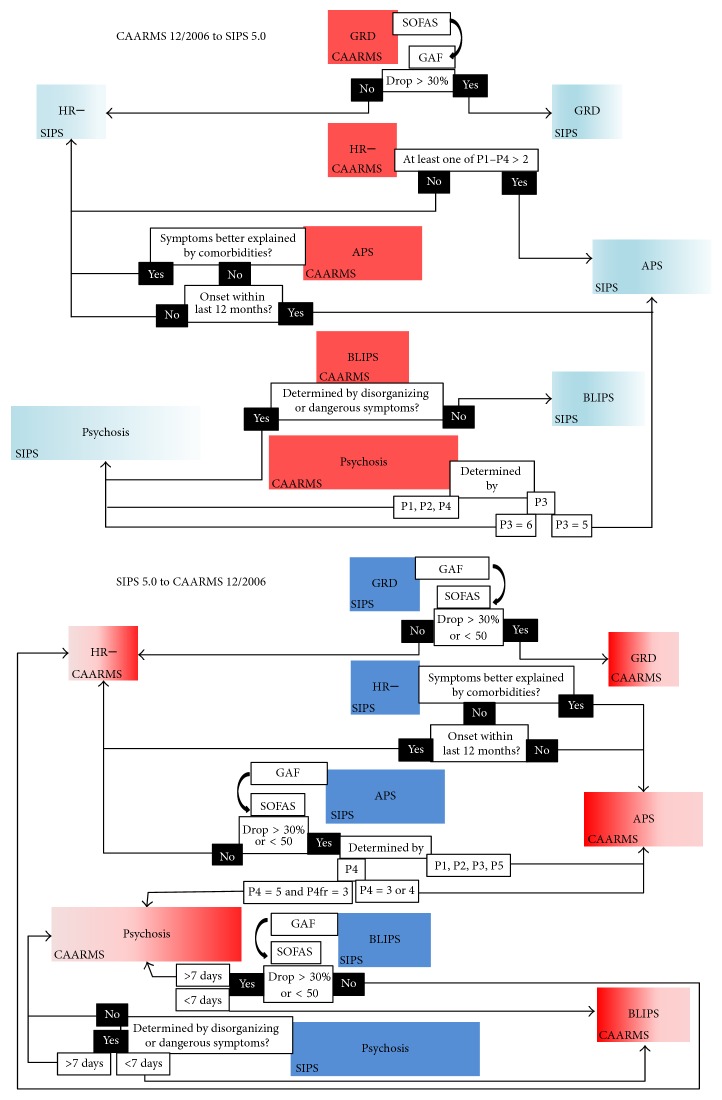
Pragmatic algorithms to convert CAARMS 12/2006-versus-SIPS 5.0 individual cases, automatized in the package CONVERT, which is appended online at https://bitbucket.org/ioppn/convert.

The pragmatic algorithm to convert individual cases across the SIPS 5.0 and the CAARMS 12/2006 is depicted in Figure 1.

The steps illustrated in Figure 1 have been fully automatized in the CONVERT tool, which is appended online (https://bitbucket.org/ioppn/convert) with further information for users. The tool was first piloted on the OASIS and CAMEO sample and it was able to correctly convert all cases between the two instruments. External validation was performed in an independent sample assessed for suspicion of UHR symptoms at the Seoul Youth Clinic (see eTable 5). For the SIPS 5.0-to-CAARMS 12/2006 CONVERT conversion, the percent overall agreement was of 92.47% (expected agreement 56.63%), kappa = 0.826 (95% CI from 0.623 to 1), PABAK = 0.85, sensitivity = 83.87%, specificity = 96.77%, and ROC area = 0.903 (95% CI from 0.833 to 0.972). For the CAARMS 12/2006-to-SIPS 5.0 CONVERT conversion, the percent overall agreement was of 98.92% (expected agreement 86.99%), kappa = 0.917 (95% CI from 0.714 to 1), PABAK = 0.98, sensitivity = 100%, specificity = 85.7%, and ROC area = 0.929 (95% CI from 0.789 to 1).

## 4. Discussion 

This is the first pilot study addressing comparability of the two psychometric instruments most frequently used to interview subjects seeking help from high-risk services for psychosis. Strengths of this study include enrolment of a relatively large sample size inclusive of UHR+, UHR−, and psychotic patients, the use of kappa analyses and equipercentile-linking methods, the development of automatized algorithms, and the use of external validation samples of different ethnic background. We found an overall substantial agreement between CAARMS 12/2006 and SIPS 5.0 in the identification of UHR subjects, with the exception of BLIPS. These findings however may be influenced by the type of recruitment strategies adopted by the high-risk services. Residual and qualitative analyses and equipercentile-linking tables provided additional comparability data. The automated conversion algorithm (CONVERT) to convert individual cases was validated in an independent sample and showed an excellent accuracy.

Our first aim was to test the diagnostic comparability of CAARMS 12/2006 versus SIPS 5.0 in a large sample of more than 200 subjects referred for psychometric diagnostic interview to high-risk services. We found an overall substantial agreement (kappa) between the CAARMS 12/2006 and SIPS 5.0. Such a substantial agreement is not completely surprising. First, the two instruments show similar psychometric parameters, such as excellent reliability properties (overall IRR agreement for the SIPS 0.95 [25], for the CAARMS 0.85 [6]). Second, independent authors not involved with the development of the two instruments argue that the development of the SIPS was influenced by the CAARMS. Specifically, they claimed that the SIPS was developed at the PRIME Clinic “to evaluate the severity of ARMS, as defined by [9].” Third, in our previous meta-analysis we found that the CAARMS and the SIPS can identify a similar proportion of true positives over time (transition risk by 31 months with the CAARMS = 27.4%, 95% CI from 24.6% to 30.4%; transition risk by 31 months with the SIPS = 28.1%, 95% CI from 25.1% to 31.3%; *p* = 0.73) [12]. Finally, in a recent meta-analysis we specifically confirmed that, in help-seeking samples, the two instruments share similar excellent prognostic accuracy in ruling out psychosis risk, with no significant differences [26]. On the basis of the above substantial agreement, our findings are the first to address the “Babylonian confusion” of UHR diagnostic interview, making it easier to overall compare the results of UHR research, which had formerly been difficult and risky [9]. Indeed, the definition of case (i.e., whether or not a subject has the condition of interest) is highly problematic across the entirety of clinical psychiatry, where no objectively assessable measures/markers/tests/exams other than clinical impression usually exist to establish the presence or absence of a given condition. Our results are thus highly relevant to permit overall meaningful comparisons of clinical, neurobiological, neurocognitive, and cost-effectiveness UHR studies worldwide, with potential beneficial impact for ongoing large-scale multicentre UHR projects such as the PRONIA (http://www.pronia.eu/), NAPLS (http://napls.commons.yale.edu/), and PSYSCAN (http://www.psyscan.eu/).

Our secondary aim was to qualitatively investigate potential discrepancies across the two instruments and to provide equipercentile-linking comparisons. The equipercentile-linking table (Table 4) suggests a close relationship between the symptoms severity and frequency scores of the CAARMS 12/2006 and SIPS 5.0, as also confirmed by equipercentile-linking analyses of SOFAS and GAF measures [27]. However, we also found some sources of disagreement, in particular, with respect to the diagnosis of BLIPS subjects. Indeed 14 out of the 25 BLIPS subjects diagnosed by the CAARMS 12/2006 were diagnosed as already psychotic by the SIPS 5.0 (eTable 4). There are significant differential operationalizations of the BLIPS across the two instruments. On one side the psychosis threshold is higher in the SIPS 5.0 than in the CAARMS 12/2006 (psychotic symptoms may last more than 7 days); on the other it is lower since the symptoms should not have urgency features [28] (“*urgency is any positive psychotic symptom that is seriously disorganizing or dangerous no matter what the duration”* (*SIPS 5.0 manual page 15* [8])). To elucidate this difference we conducted a qualitative analysis of the medical records of these 14 subjects. We confirmed that all of them were presenting with disorganized or dangerous symptoms (see definition in Table 2), which were meeting BLIPS criteria under the CAARMS 12/2006 (eTable 3) while at the same time being regarded as over threshold for psychosis with the SIPS 5.0. Since South London has one of the highest rates of psychosis in the world [17], BLIPS subjects alone represent about 9% of OASIS patients, with an additional 9% meeting conjointly APS or GRD criteria (Figure 6 from [17]). Conversely, operationalization differences in BLIPS duration (i.e., less than 7 days on the CAARMS 12/2006) affected only 4 subjects in our database (eTable 3). This may be partially due to the fact that our referrers are trained to refer only BLIPS subjects defined by the CAARMS 12/2006 criteria. Similarly, differences on psychosis threshold in the Perceptual Abnormalities subscale (i.e., severity of 6 on the SIPS 5.0 and 5 on the CAARMS 12/2006) affected only 6 subjects in our database (eTable 3).

Conversely, operationalization differences of the APS, which includes four domains (P1–P4) in the CAARMS 12/2006 and five in the SIPS 5.0 (P1–P5), did not play a significant role (eTable 3). It is possible to speculate that the additional SIPS 5.0 domain, Grandiose Ideas, with manic or hypomanic features is not particularly frequent or severe in UHR subjects (4 subjects only had higher scores on SIPS 5.0 P3 as compared to SIPS 5.0 P2). Also, operationalization differences in APS onset criteria did not impact the overall consistency of the diagnostic interview for APS across the two instruments. This is somewhat surprising giving that CAARMS 12/2006 permits positive symptoms to qualify even if they are no longer present in the past month, whereas SIPS 5.0 requires them to be present in the past month. Also, the functional decline criterion (i.e., SOFAS drop), which is explicitly used in the CAARMS 12/2006 but not in the SIPS 5.0, did not impact the diagnostic agreement (see limitations below). This finding however is also supported by our recent meta-analysis showing that both SIPS-defined and CAARMS-defined UHR samples display consistent baseline functional impairments as compared to matched controls [20]. Similarly, there was no effect for the assessment of differential diagnoses associated with comorbidities [29] between the CAARMS 12/2006 (which does not consider comorbidities) and SIPS 5.0 (in which the UHR+ diagnosis should not be made if the symptoms are better explained by other comorbid disorders; this affected only 5 subjects in our database; see eTable 3). However, the notion “better explained” is poorly coded and may therefore be subject to arbitrary clinical judgment: no studies have ever addressed the impact of this criterion on subjects identified by the SIPS. Because of this, comorbid disorders may be highly prevalent in both the CAARMS 12/2006 and the SIPS 5.0. Indeed in our meta-analysis specifically investigating comorbid affective disorders in UHR subjects, we found a similar prevalence of anxiety or depressive disorders in SIPS [30, 31] and CAARMS [32] studies.

Our third aim was to develop an automated algorithm to convert individual cases and to validate it in an external sample. Therefore, on the basis of the CAARMS 12/2006 and SIPS 5.0 differences in operationalizations detailed in Tables 1 and 2, we were finally able to develop and propose a pragmatic algorithm to convert the main clinical outcomes across the two instruments (see Figure 1). This algorithm has been implemented in the CONVERT tool, which has been made freely available for the use of future researchers and clinicians and externally validated in an independent sample. We found that CONVERT performed in the excellent range of accuracy in both directions: the ROC area was 0.903 for the SIPS 5.0-to-CAARMS 12/2006 conversion and 0.929 for the CAARMS 12/2006-to-SIPS 5.0 conversion. The ROC area serves as a global measure of test performance and values in the range of 0.9–1 are considered excellent, between 0.8 and 0.9 very good, between 0.7 and 0.8 good, between 0.6 and 0.7 sufficient, and between 0.5 and 0.6 bad [33]. Of relevance, CONVERT was developed on a priori differences between CAARMS 12/2006 and SIPS 5.0 operationalizations (Tables 1 and 2), and its performance was tested against an external validation sample that was characterized by a different ethnic background. Because of this, we expect that CONVERT could perform well in other UHR samples and we hope that it will facilitate data merging across UHR sites employing different diagnostic instruments. This may support large-scale multicentre UHR analyses across PRONIA, PSYSCAN, or NAPLS or replication studies to consolidate the current UHR findings.

This study had limitations. First, we did not perform a follow-up. Second, the type of recruitment adopted in OASIS and CAMEO services may have impacted the observed substantial agreement between CAARMS 12/2006 and SIPS 5.0. For example, the close link of the OASIS or CAMEO with the local first-episode services may have increased the proportion of disorganizing/dangerous BLIPS or the clinical composition of subjects referred for UHR assessment to our services [34]. Also, our referrers underwent a long-standing training to identify and signpost subjects meeting the functional deterioration criterion according to the CAARMS 12/2006 intake criteria. Therefore, it is possible that UHR patients meeting SIPS 5.0 criteria (e.g., with attenuated psychotic symptoms but without functional deterioration) may have been undetected by referrers and not assessed at all by our teams, inflating the observed agreement. Indeed, when the CAARMS 12/2006 was compared with the SIPS 5.0 in other epidemiological samples of non-help-seeking subjects, significant differences between the two instruments were observed [35] (the use of UHR instruments in non-help-seeking samples is not recommended however [11]). There is also recent meta-analytical evidence indicating that samples referred to high-risk services are highly heterogeneous and that their actual composition may reflect the type of outreach campaigns adopted [36, 37]. Future studies should investigate the likely possibility of a lower agreement between CAARMS 12/2006 and SIPS 5.0 in high-risk services employing SIPS-based outreach campaigns. Third, our procedure involving a unique rater scoring both instruments in an uncontrolled order may have significantly inflated agreement across instruments. However, assessing subjects referred for suspicion of UHR symptoms at the time of the first contacts with high-risk services (who may be already psychotic or eventually deemed not at risk of psychosis) with independent raters poses severe logistic difficulties for the patients. It may also paradoxically create additional biases because the most severe patients may be more likely to decline lengthy assessments. To control for this we performed an independent analysis in a subset of patients (*n* = 21) assessed with independent raters and we confirmed that magnitude of agreement remained substantial (see results in eMethod 1). Interrater reliability of the original instruments has been investigated in even smaller samples (*n* = 14) [25]. Fourth, given the differences in the two instruments' development itself [10], our findings and the CONVERT tool may not be applied to older versions of the instruments. Fifth, psychometric reconciliation of the CAARMS versus SIPS is not sufficient to mitigate the differences between the various UHR research teams. Differences remain between the characteristics of the basic population, the recruitment of patients, the follow-up, and the specific treatments provided [9].

## 5. Conclusions

There is overall substantial diagnostic agreement between the CAARMS 12/2006 and SIPS 5.0 towards identification of UHR subjects. Disagreement was mostly due to differential operationalization of BLIPS. However, type of recruitment strategies may have inflated the observed agreement and future studies should repeat these analyses in high-risk services adopting different outreach campaigns. The conversion algorithm, CONVERT, had excellent performance characteristics even in samples of different ethnic background. The results of the current investigation may be highly relevant to the field, as they may inform future multicentre studies as well as international consensus conferences aiming at standardizing the UHR diagnostic interview.

## Supplementary Material

The supplementary materials include information on the differences in the psychopathological dimensions of attenuated psychotic symptoms between the CAARMS 12/2006 and SIPS 5.0 (eTable 1). In the eMethods we provided details on the training procedure and the interrater reliability of the OASIS and CAMEO clinicians and on the equipercentile equating procedure to convert the severity and frequency of symptoms belonging to similar psychopathological constructs under the CAARMS 12/2006 and the SIPS 5.0. eFigure 1 is adapted from [19] and depicts Relative functional deterioration in subjects at Ultra High Risk, compared to Psychosis. We compared sociodemographic characteristics of referrals across OASIS and CAMEO in eTable 2. eTable 3 shows the post-hoc analyses investigating the agreement across the two psychometric instruments. A qualitative analysis of the psychopathological characteristics of 14 patients diagnosed as BLIPS by the CAARMS 12/2006, but as psychotic by the SIPS 5.0 is presented in eTable 4. Finally, eTable 5 describes the results of the validation of CONVERT in the external independent sample of 93 subjects assessed with both CAARMS 12/2006 and SIPS 5.0 at the Seoul Youth Clinic.

## Figures and Tables

**Table:** (a) Rating system

	SIPS *version 5.0* [8]	CAARMS *version 12/2006* [16]
Subscales	P1: Unusual Thought Content/Delusional Ideas P2: Suspiciousness/Persecutory Ideas P3: Grandiose Ideas P4: Perceptual Abnormalities/Hallucinations P5: Disorganized Communication	P1: Unusual Thought Content P2: Non-Bizarre Ideas P3: Perceptual Abnormalities P4: Disorganized Speech

Frequency	1: at least several min per d at least 1/mo 2: several min/d at least once/wk in the past mo 3: at least 1 h/d for at least 4 d/wk over 1 mo	0: absent 1: less than 1/mo 2: 1/mo to 2/wk, <1 h per occasion 3: 1/mo to 2/wk, >1 h per occasion, OR 3 to 6/wk, <1 h per occasion 4: 3 to 6/wk, > 1 h per occasion, OR daily, <1 h per occasion 5: daily, >1 h per occasion, OR several times/d 6: continuous

Substance use	Exclusion criterion if strongly intertwined with symptoms	0: no relation to substance use noted 1: occurs in relation to substance use and at other times as well 2: noted only in relation to substance use

Distress	Subjective qualifier Not used to determine an individual's UHR status	Rated on scale 0–100 Not used to determine an individual's UHR status

**Table:** (b) Attenuated psychotic symptoms

	Attenuated positive symptom psychosis-risk syndrome SIPS *version 5.0* [8]	Attenuated psychosis group CAARMS *version 12/2006* [16]
Inclusion criteria	Severity score of 3–5 on at least one of P1–P5 *PLUS* Frequency score of 2 on P1, P2, P3, P4, and/or P5	*Subthreshold intensity* Severity score of 3–5 on P1, 3–5 on P2, 3-4 on P3, and/or 4-5 on P4 *PLUS* Frequency score of 3–6 on P1, P2, P3, and/or P4 *Subthreshold frequency* Severity score of 6 on at least one of P1, P2, and P4 and/or 5-6 on P3 *PLUS* Frequency score of 3 on P1, P2, P3, and/or P4

Onset	Symptoms should have begun within the past year *OR* currently rate one or more scale points higher compared to 12 months before Symptoms that occurred over the past month only are rated	Symptoms should have been present in the previous 12 mo *AND* for not longer than 5 y

Level of functioning	No social/occupational dysfunction requirement	30% drop in SOFAS score from premorbid level, sustained for a mo, within the past 12 mo *OR* SOFAS score <50 for the past 12 mo or more

Exclusion criteria	Symptoms are strongly intertwined temporally with substance use episodes (substance-induced psychosis may be considered)	Symptoms occur only during peak intoxication from a substance known to be associated with psychotic experiences (e.g., hallucinogens, amphetamines, and cocaine)
	Symptoms are better accounted for by another DSM diagnosis	—
	Past psychosis ruled in according to information obtained through the initial screen and evaluated using the POPS	The person has had a previous psychotic episode (treated or untreated)

**Table:** (c) Brief limited intermittent psychotic symptoms

	Brief intermittent psychotic symptom psychosis-risk syndrome SIPS *version 5.0* [8]	Brief limited intermittent psychotic symptoms group CAARMS *version 12/2006* [16]
Inclusion criteria	Severity score of 6 on at least one of P1–P5 *PLUS* Frequency score of 1 on P1, P2, P3, P4, and/or P5	Severity score of 6 on at least one of P1, P2, and P4 and/or 5-6 on P3 *PLUS* Frequency score of 4–6 on P1, P2, P3, and/or P4

Onset	Symptoms should have reached a psychotic level of intensity in the previous 3 mo	Symptoms should have been present in the previous 12 mo and for not longer than 5 y

Duration	Up to 3 mo	Up to 7 d

Level of functioning	No social/occupational dysfunction requirement	30% drop in SOFAS score from premorbid level, sustained for a mo, within the past 12 mo *OR* SOFAS score <50 for the past 12 mo or more

Exclusion criteria	Symptoms are strongly intertwined temporally with substance use episodes (substance-induced psychosis may be considered)	Symptoms occur only during peak intoxication from a substance known to be associated with psychotic experiences (e.g., hallucinogens, amphetamines, and cocaine)
	Symptoms are better accounted for by another DSM diagnosis	—
	Past psychosis ruled in according to information obtained through the initial screen and evaluated using the POPS	The person has had a previous psychotic episode (treated or untreated)
	Symptoms are seriously disorganizing and dangerous	—
	—	Symptoms do not resolve spontaneously (without antipsychotic medication)

**Table:** (d) Genetic risk and deterioration syndrome

	Genetic risk and deterioration psychosis-risk syndrome SIPS *version 5.0* [8]	Vulnerability group CAARMS *version 12/2006* [16]
Inclusion criteria	The patient meets criteria for Schizotypal Personality Disorder *OR* The patient has a first-degree relative with a psychotic disorder	Schizotypal Personality Disorder in identified patient *OR* Family history of psychosis in a first-degree relative

Level of functioning	30% drop in GAF score over the last mo as compared to 12 mo before	30% drop in SOFAS score from premorbid level, sustained for a mo, within the past 12 mo *OR* SOFAS score <50 for the past 12 mo or more

CAARMS, Comprehensive Assessment of At-Risk Mental States; d, day; GAF, Global Assessment of Functioning; h, hour; min, minute; mo, month; SIPS, Structured Interview for Psychosis-Risk Syndrome; SOFAS, Social and Occupational Functioning Assessment Scale; UHR, ultra high risk; wk, week.

**Table 2:** Psychosis threshold: similarities and differences between the SIPS 5.0 and the CAARMS 12/2006.

	Presence of psychotic syndrome SIPS *version 5.0*	Psychosis threshold CAARMS *version 12/2006*
Inclusion criteria	Severity score of 6 on at least one of P1–P5 *PLUS* Frequency score of 3 on P1, P2, P3, P4, and/or P5	Severity score of 6 on at least one of P1, P2, and P4 and/or 5-6 on P3 *PLUS* Frequency score of 4–6 on P1, P2, P3, and/or P4

Urgency	Symptoms are seriously disorganizing and dangerous (a)	—

CAARMS, Comprehensive Assessment of At-Risk Mental States; SIPS, Structured Interview for Psychosis-Risk Syndrome.

(a) “‘Dangerous' is taken to mean physically dangerous, for example, risk of death or serious physical injury, and ‘disorganizing' means potentially psychosocially dangerous, for example, risk of seriously damaging work relations, social relations, family relations, or personal dignity.” Personal communication from the authors of the SIPS, published with permission (see Acknowledgments).

**Table 3:** Diagnostic comparison between CAARMS 12/2006 and SIPS 0.5 outcomes in subjects seeking help from high-risk services (*p* < 0.001).

			SIPS outcome			
			UHR−	UHR+	Psychotic	Total
CAARMS outcome	UHR−	Count	51	0	0	51
		%	24.1%	0.0%	0.0%	24.1%
		Adj Res	13.5	−7.8	−4.8	
	UHR+	Count	5	92	14	111
		%	2.4%	91.1%	6.6%	52.4%
		Adj Res	−7.7	10.8	−4.5	
	Psychotic	Count	1	9	40	50
		%	0.5%	4.2%	18.9%	23.6%
		Adj Res	−4.5	−4.8	10.1	
	Total	Count	57	101	54	212
		%	29.6%	47.6%	25.5%	100.0%

CAARMS, Comprehensive Assessment of At-Risk Mental States; SIPS, Structured Interview for Psychosis-Risk Syndrome; UHR, ultra high risk; Adj Res, adjusted residuals; adjusted residuals lower than −3.29 or greater than 3.29 indicate that the number of cases in that cell is significantly larger or small than expected under the null hypothesis at *p* < 0.001 corrected for multiple comparisons. No cells have expected count less than 5. The minimum expected count is 12.74.

**Table 4:** Equipercentile-linking table comparing symptoms severity and frequency across CAARMS 12/2006 and SIPS 5.0 in the OASIS and CAMEO sample.

Domain	Severity scores				Frequency scores			
	CAARMS to	SIPS	SIPS to	CAARMS	CAARMS to	SIPS	SIPS to	CAARMS
P1 CAARMS to P1 SIPS & P1 SIPS to P1 CAARMS	0	0.012	0	0.011	0	0.019	0	0.017
	1	1.092	1	0.916	1	0.718	1	1.690
	2	2.212	2	1.816	2	1.163	2	3.321
	3	3.258	3	2.759	3	1.781	3	5.282
	4	4.180	4	3.799	4	2.481		
	5	5.019	5	4.976	5	2.899		
	6	5.965	6	6.033	6	3.289		

P2 CAARMS to P2 SIPS	0	0.026			0	0.028		
	1	1.059			1	0.602		
	2	2.234			2	1.001		
	3	3.216			3	1.669		
	4	4.105			4	2.406		
	5	5.956			5	2.861		
	6	5.961			6	3.271		

P2 CAARMS to P3 SIPS	0	0.041			0	0.004		
	1	0.124			1	0.027		
	2	0.178			2	0.071		
	3	0.112			3	0.106		
	4	0.297			4	0.237		
	5	0.464			5	0.469		
	6	2.692			6	2.462		

P2/P3 SIPS^*∗*^ to P2 CAARMS			0	0.007			0	0.062
			1	0.919			1	1.779
			2	1.778			2	3.541
			3	2.735			3	5.478
			4	3.806				
			5	4.991				
			6	6.025				

P3 CAARMS to P4 SIPS & P4 SIPS to P3 CAARMS	0	0.012	0	0.013	0	0.007	0	0.007
	1	0.893	1	1.112	1	0.625	1	1.984
	2	1.891	2	2.106	2	1.001	2	3.347
	3	2.953	3	3.045	3	1.701	3	4.899
	4	3.944	4	4.059	4	2.537		
	5	4.861	5	5.153	5	3.034		
	6	5.876	6	6.099	6	3.530		

P4 CAARMS to P5 SIPS & P5 SIPS to P4 CAARMS	0	0.067	0	0.079	0	0.080	0	0.095
	1	0.855	1	1.126	1	0.594	1	1.743
	2	1.981	2	2.017	2	1.133	2	3.612
	3	3.032	3	2.968	3	1.666	3	5.234
	4	4.064	4	3.936	4	2.193		
	5	5.158	5	4.844	5	2.798		
	6	6.090	6	5.889	6	3.472		

P1–P4 on the CAARMS and P1–P5 on SIPS are defined in Table 1; ^*∗*^using the highest score across P2 and P3 SIPS. For analytical purposes in this table the SIPS frequency was coded as follows: 1: ≥1 h/d, ≥4 d/wk; 2: ≥several minutes/d, ≥1x/mo; 3: ≥1x/wk; 0: none of the above.

## Acronyms and Abbreviations

UHR+: At ultra high risk of psychosis
UHR−: Not at ultra high risk of psychosis
CAARMS: Comprehensive Assessment of At-Risk Mental States
SIPS: Structured Interview for Psychosis-Risk Syndrome
APS: Attenuated psychotic symptoms
BLIPS: Brief limited intermittent psychotic symptoms
GRD: Genetic Risk and Deterioration syndrome
ROC: Receiver Operating Characteristic
fr: Frequency
d: Day
GAF: Global Assessment of Functioning
h: Hour
min: Minute
mo: Month
SOFAS: Social and Occupational Functioning Assessment Scale
wk: Week.

## References

[1] Addington J., Stowkowy J., Weiser M. (2015). Screening tools for clinical high risk for psychosis. *Early Intervention in Psychiatry*.

[2] Fusar-Poli P., Carpenter W. T., Woods S. W., McGlashan T. H. (2014). Attenuated psychosis syndrome: ready for DSM-5.1?. *Annual Review of Clinical Psychology*.

[3] Stafford M. R., Jackson H., Mayo-Wilson E., Morrison A. P., Kendall T. (2013). Early interventions to prevent psychosis: systematic review and meta-analysis. *BMJ*.

[4] Jackson H. J., McGorry P. D., McKenzie D. (1994). The reliability of DSM-III prodromal symptoms in first-episode psychotic patients. *Acta Psychiatrica Scandinavica*.

[5] Yung A. R., McGorry P. D., McFarlane C. A., Jackson H. J., Patton G. C., Rakkar A. (1996). Monitoring and care of young people at incipient risk of psychosis. *Schizophrenia Bulletin*.

[6] Yung A. R., Yuen H. P., McGorry P. D. (2005). Mapping the onset of psychosis: the comprehensive assessment of At-Risk Mental States. *Australian and New Zealand Journal of Psychiatry*.

[7] Miller T. J., McGlashan T. H., Woods S. W. (1999). Symptom assessment in schizophrenic prodromal states. *Psychiatric Quarterly*.

[8] McGlashan T., Walsh B., Woods S. (2010). *The Psychosis-Risk Syndrome: Handbook for Diagnosis and Follow-Up*.

[9] Daneault J.-G., Stip E., Villeneuve M. (2013). Genealogy of instruments for prodrome evaluation of psychosis. *Frontiers in Psychiatry*.

[10] Schultze-Lutter F., Schimmelmann B. G., Ruhrmann S., Michel C. (2013). ‘A rose is a rose is a rose’, but at-risk criteria differ. *Psychopathology*.

[11] Schultze-Lutter F., Michel C., Schmidt S. J. (2015). EPA guidance on the early detection of clinical high risk states of psychoses. *European Psychiatry*.

[12] Fusar-Poli P., Bonoldi I., Yung A. R. (2012). Predicting psychosis: meta-analysis of transition outcomes in individuals at high clinical risk. *Archives of General Psychiatry*.

[13] Schultze-Lutter F., Schimmelmann B. G., Ruhrmann S. (2011). The near babylonian speech confusion in early detection of psychosis. *Schizophrenia Bulletin*.

[14] Kapur S., Phillips A. G., Insel T. R. (2012). Why has it taken so long for biological psychiatry to develop clinical tests and what to do about it?. *Molecular Psychiatry*.

[15] Dorans N. J., Holland P. W. (2000). Population invariance and the equatability of tests: basic theory and the linear case. *Journal of Educational Measurement*.

[16] Yung A. R., Phillips L. J., Yuen H. P., McGorry P. D. (2006). *Comprehensive Assessment of at Risk Mental State*.

[17] Fusar-Poli P., Byrne M., Badger S., Valmaggia L. R., McGuire P. K. (2013). Outreach and support in South London (OASIS), 2001–2011: ten years of early diagnosis and treatment for young individuals at high clinical risk for psychosis. *European Psychiatry*.

[18] Zimbrón J., Ruiz de Azúa S., Khandaker G. M. (2013). Clinical and sociodemographic comparison of people at high-risk for psychosis and with first-episode psychosis. *Acta Psychiatrica Scandinavica*.

[19] Kwon J. S., Byun M. S., Lee T. Y., An S. K. (2012). Early intervention in psychosis: insights from Korea. *Asian Journal of Psychiatry*.

[20] Fusar-Poli P., Rocchetti M., Sardella A. (2015). Disorder, not just state of risk: meta-analysis of functioning and quality of life in people at high risk of psychosis. *British Journal of Psychiatry*.

[21] Vannest K. J., Parker R. I., Gonen O. (2011). *Single Case Research: Web Based Calculators for SCR Analysis*.

[22] Byrt T., Bishop J., Carlin J. B. (1993). Bias, prevalence and kappa. *Journal of Clinical Epidemiology*.

[23] Landis J. R., Koch G. G. (1977). The measurement of observer agreement for categorical data. *Biometrics*.

[24] Albano A. (2014). *Equate: Observed-Score Linking and Equating*.

[25] Miller T. J., McGlashan T. H., Rosen J. L. (2003). Prodromal assessment with the structured interview for prodromal syndromes and the scale of prodromal symptoms: predictive validity, interrater reliability, and training to reliability. *Schizophrenia Bulletin*.

[26] Fusar-Poli P., Cappucciati M., Rutigliano G. (2015). At risk or not at risk? A meta-analysis of the prognostic accuracy of psychometric interviews for psychosis prediction. *World Psychiatry*.

[27] Samara M. T., Engel R. R., Millier A., Kandenwein J., Toumi M., Leucht S. (2014). Equipercentile linking of scales measuring functioning and symptoms: examining the GAF, SOFAS, CGI-S, and PANSS. *European Neuropsychopharmacology*.

[28] Fusar-Poli P., Van Os J. (2013). Lost in transition: setting the psychosis threshold in prodromal research. *Acta Psychiatrica Scandinavica*.

[29] Modinos G., Allen P., Frascarelli M. (2014). Are we really mapping psychosis risk? Neuroanatomical signature of affective disorders in subjects at ultra high risk. *Psychological Medicine*.

[30] Ruhrmann S., Schultze-Lutter F., Salokangas R. K. R. (2010). Prediction of psychosis in adolescents and young adults at high risk: results from the prospective European prediction of psychosis study. *Archives of General Psychiatry*.

[31] Woods S. W., Addington J., Cadenhead K. S. (2009). Validity of the prodromal risk syndrome for first psychosis: findings from the North American Prodrome Longitudinal Study. *Schizophrenia Bulletin*.

[32] Fusar-Poli P., Nelson B., Valmaggia L., Yung A. R., McGuire P. K. (2014). Comorbid depressive and anxiety disorders in 509 individuals with an at-risk mental state: impact on psychopathology and transition to psychosis. *Schizophrenia Bulletin*.

[33] Obuchowski N. A., Lieber M. L., Wians F. H. (2004). ROC curves in clinical chemistry: uses, misuses, and possible solutions. *Clinical Chemistry*.

[34] Rietdijk J., Klaassen R., Ising H. (2012). Detection of people at risk of developing a first psychosis: comparison of two recruitment strategies. *Acta Psychiatrica Scandinavica*.

[35] Kelleher I., Murtagh A., Molloy C. (2012). Identification and characterization of prodromal risk syndromes in young adolescents in the community: a population-based clinical interview study. *Schizophrenia Bulletin*.

[36] Fusar-Poli P., Schultze-Lutter F., Cappucciati M. (2016). The dark side of the moon: meta-analytical impact of recruitment strategies on risk enrichment in the clinical high risk state for psychosis. *Schizophrenia Bulletin*.

[37] Fusar-Poli P., Schultze-Lutter F., Addington J. (2016). Intensive community outreach for those at ultra high risk of psychosis: dilution, not solution. *The Lancet Psychiatry*.

