# Supplementary material for: Towards a Standard Psychometric Diagnostic Interview for Subjects at Ultra High Risk of Psychosis: CAARMS versus SIPS
Source: Psychiatry J. 2016 May 30;2016:7146341. doi: 10.1155/2016/7146341 (PMC4904115; doi:10.1155/2016/7146341)
Supplement: Supplementary file 1 — The supplementary materials include information on the differences in the psychopathological dimensions of attenuated psychotic symptoms between the CAARMS 12/2006 and SIPS 5.0 (eTable 1). In the eMethods we provided details on the training procedure and the interrater reliability of the OASIS and CAMEO clinicians and on the equipercentile equating procedure to convert the severity and frequency of symptoms belonging to similar psychopathological constructs under the CAARMS 12/2006 and the SIPS 5.0. eFigure 1 is adapted from [19] and depicts Relative functional deterioration in subjects at Ultra High Risk, compared to Psychosis. We compared sociodemographic characteristics of referrals across OASIS and CAMEO in eTable 2. eTable 3 shows the post-hoc analyses investigating the agreement across the two psychometric instruments. A qualitative analysis of the psychopathological characteristics of 14 patients diagnosed as BLIPS by the CAARMS 12/2006, but as psychotic by the SIPS 5.0 is presented in eTable 4. Finally, eTable 5 describes the results of the validation of CONVERT in the external independent sample of 93 subjects assessed with both CAARMS 12/2006 and SIPS 5.0 at the Seoul Youth Clinic. [file 7146341.f1.pdf]

eTable 1. *Psychopathological dimensions of attenuated psychotic symptoms investigated by the diagnostic subscales of the CAARMS 12/2006 (P1-P4) and SIPS 5.0 (P1-P5)*

**CAARMS** ***Version 12/2006[1]***	**SIPS** ***Version 5.0[2]***
**P1. UNUSUAL THOUGHT CONTENT**	**P1.UNUSUAL THOUGHT CONTENT/DELUSIONAL IDEA**
Delusional Mood and Perplexity ("Non Crystallized Ideas")	Perplexity and Delusional Mood
	Overvalued Beliefs
Ideas of Reference	Non-persecutory Ideas of Reference
Bizarre Ideas ('Crystallized Ideas')	First Rank Symptoms
• Made thoughts, feelings, impulses	
• Somatic Passivity	
• Thought Insertion	
• Thought Withdrawal	
• Thought Broadcasting	
• Thoughts Being Read	
**P2. NON-BIZARRE IDEAS**	
Non-bizarre Ideas ("Crystallized Ideas")	Other Unusual Thoughts/Delusional Ideas
• Ideas of Guilt	• Somatic Ideas
• Somatic Ideas	• Nihilistic Ideas
• Nihilistic Ideas	• Ideas of Guilt
• Religious Ideas	
• Erotomanic Ideas	**P2. SUSPICIOUSNESS/PERSECUTORY IDEAS**
• Jealous Ideas	**P3. GRANDIOSE IDEAS**
• Suspiciousness, Persecutory Ideas	
• Grandiose Ideas	

P3. PERCEPTUAL ABNORMALITIES	P4. PERCEPTUAL ABNORMALITIES/HALLUCINATIONS
	Perceptual Distortions, Illusions, Hallucinations
Visual Changes<br>• Distortions, illusions<br>• Hallucinations	Visual Distortions, Illusions, Hallucinations
Auditory Changes<br>• Distortions, illusions<br>• Hallucinations	Auditory Distortions, Illusions, Hallucinations
Olfactory Changes<br>• Distortions, illusions<br>• Hallucinations<br>Gustatory Changes<br>• Distortions, illusions<br>• Hallucinations	Olfactory and Gustatory Distortions, Illusions, Hallucinations
Tactile Changes<br>• Distortions, illusions<br>• Hallucinations<br>Somatic Changes<br>• Distortions, illusions<br>• Hallucinations	Somatic Distortions, Illusions, Hallucinations
**P4. DISORGANIZED SPEECH**	**P5. DISORGANIZED COMMUNICATION**
Subjective Change<br>Objective Rating of Disorganized Speech	Communication Difficulties

eMethod 1. *Training and inter rater reliability procedure*

Six raters, three from the OASIS in Lambeth and Southwark (SB, team leader; MC, psychiatrist; SK, psychiatrist), two from the OASIS in Lewisham (RP, psychiatrist; VB, psychiatrist) and one from the CAMEO (QB, team leader) conducted the diagnostic interviews. They were weekly supervised by senior consultant psychiatrists (PFP, OASIS in Lambeth and Southwark; IB, OASIS in Lewisham; JP, CAMEO). The average experience with UHR assessments for raters and supervisors was of 2.83 (SD 2.1) years and of 7.3 (SD 2.31) years respectively. Both services use the same psychometric package, which is based on the CAARMS Version 12/2006[1]. CAARMS training has been previously established as standard procedure across the two teams in two studies funded by the Medical Research Council and European Union (data under preparation). In a first step, raters used the ORYGEN training package (https://orygen.org.au/Skills-Knowledge/Resources/DVD-Video-Audio/CAARMS-DVD), which includes instructional DVD and supporting workbook. They learnt how to use the CAARMS via case scenario interviews and self-assessment practice vignettes. Specifically, these online resources were made available by the EU-GEI UHR study (http://www.eu-gei.eu/training-videos); at the end of the online training raters received a certificate of approval for the clinical use of the CAARMS. In a second step, regular training sessions (three times a year) were organized at the clinical teams for further practice with local case series. These sessions were usually led by expert clinicians (e.g. SB and PFP) with more than 10 years experience in the use of the instrument. Finally, weekly group and individual supervisions were offered to the raters by senior clinicians (PFP, IB, JP).

For the purposes of the current study, the responsible clinicians were requested to additionally score the SIPS Version 5.0***[2]***. The raters were specifically trained on the use of the SIPS by employing the official manual***[2]***. Additional training sessions focused on the differences between the two instruments as illustrated in Table 1, Table 2 and eTable 1. These sessions were complemented by practice with case vignettes taken from the local clinical caseload. The authors of the SIPS were

contacted as independent external advisors to seek further clarification when needed (see Table 2 and acknowledgments).

To estimate the risk that the same rater would inflate the agreement we further tested the kappa in a subset of subjects rated by independent raters (n=21). Each rater was usually requested to score both instruments (because of logistic difficulty only in a subset of cases it was possible to have two independent raters, see limitations). The inter rater reliability (IRR) was estimated with weighted kappa for ordinal variables, which is similar to the intra class coefficient (ICC) estimated from a two-way random effects ANOVA[3], by using the kappa2 STATA module which allows IRR when there are two ore more unique raters and two or more ratings. The IRR results are appended in the table below here

Inter Rater Reliability (IRR) for CAARMS and SIPS

Diagnostic interview instrument	K
CAARMS	
Diagnostic outcome	0.902
P1 Disorder of thought content	0.915
P2 Non-bizarre ideas	0.863
P3 Perceptual abnormalities	0.864
P4 Disorganized speech	0.875
SIPS	
Diagnostic outcome	0.854
P1 Unusual thought content/Delusional Idea	0.923
P2 Suspiciousness/Persecutory Ideas	0.822
P3 Grandiose Ideas	0.871
P4 Perceptual abnormalities/Hallucinations	0.867
P5 Disorganized communication	0.815

K, weighted kappa; sample size=21

eMethod 2. *Equipercentile equating details*

The term linking refers to the bringing together of test forms which have not been created according to the same specifications; for example, forms which differ in length or content. In this approach, the linked scales are considered similar but not interchangeable, and are related to one another via a linking function[4]. Specifically, we used equipercentile linking method in a single-group design, where one group, sampled from the target population T, takes two different test forms X and Y. Any differences in the score distributions on X and Y are thus attributed entirely to the test forms themselves, as group ability is assumed to be constant; thus, if the distributions are not the same, it is because the test forms differs[4]. Smoothing methods (loglinear presmoothing)[4] are typically used to reduce irregularities due to sampling error in either the score distributions or the equipercentile-linking function itself[4]. Equipercentile-linking has been successfully used in clinical psychiatry to link scales with different characteristics such as the PANSS, BPRS and CGI[5, 6]. Equipercentile-linking was applied to link subscales investigating similar psychopatholgical constructs featuring attenuated positive psychotic symptoms: CAARMS P1 to SIPS P1 and vice versa (disorder of thought content and delusional ideas), CAARMS P2 to SIPS P2 (cristallized and persecutory ideas), CAARMS P3 to SIPS P4 and vice versa (perceptual abnormalities), CAARMS P4 to SIPS P5 and vice versa (disorganized speech and communication)(see eTable 1). Since the SIPS P3 (grandiose ideas) is included in the CAARMS P2 subscale (cristallized ideas), we estimated the CAARMS P2 to SIPS P3 conversion scores (see eTable 1) and converted the highest score across P2 and P3 SIPS to CAARMS P2.

eFigure 1. *Relative functional deterioration in subjects at Ultra High Risk, compared to Psychosis.* Baseline mean GAF scores in studies comparing High Risk with Healthy Comparisons and Psychosis, adapted from[7]. Data are based on absolute mean GAF scores of the three groups (these values need to be considered cautiously as not direct computation of mean difference between Healthy Comparisons and Psychosis was performed at meta-analytical level). Legend: GAF, global assessment of functioning.

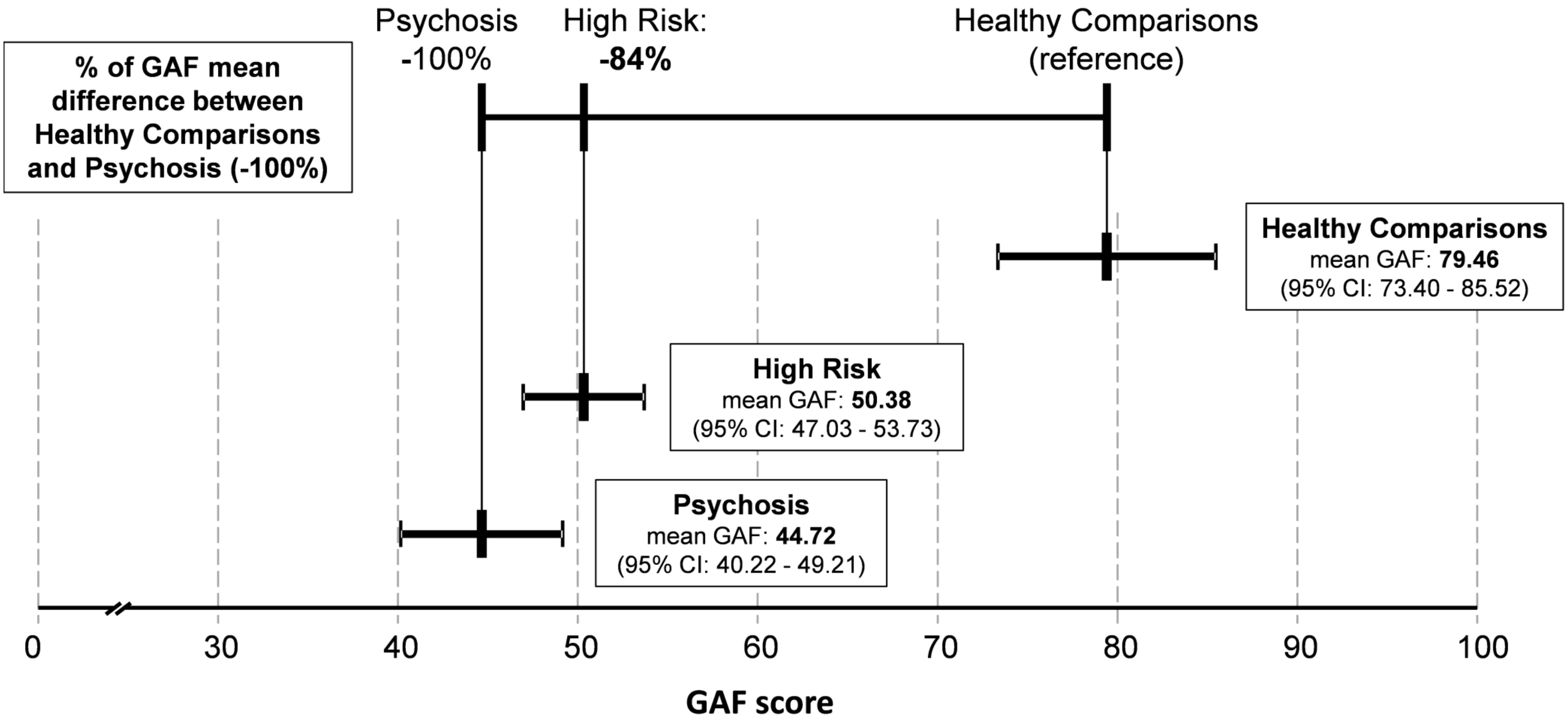

eTable 2. *Sociodemographic baseline characteristics of referrals to OASIS and CAMEO high risk services.*

	OASIS (n=124; 58,5%)	CAMEO (n=88; 41,5%)	Total (n= 212)
Age in Yrs. (Mean ± SD)	24,1 ± 4,9	21,9 ± 4,2	23,1 ± 4,8
Gender (% male)	66,1%	54,5%	61,3%
Ethnicity			
White British	27,9%	89,4%	53,1%
Black British	37,7%	2,4%	23,2%
African	6,6%	0,0%	3,9%
South American	1,6%	0,0%	1,0%
Asian	9,8%	3,5%	7,2%
Other White	16,4%	4,7%	11,6%
Psychiatric Comorbidities[a]			
None	65,0%	82,8%	72,4%
Axis I	30,9%	16,1%	24,8%
Axis II	4,1%	1,1%	2,8%

a) Based on the Structured Clinical Interview for DSM-IV (SCID-I and SCID-II)

eTable 3. *Residual analysis: CAARMS vs SIPS*

Table 2A.			SIPS Outcome					
			UHR-	BLIPS	APS	GRD	Psychotic	Total
CAARMS Outcome	UHR-	Count	51	0	0	0	0	51
		Adjusted Residual	13,5	-2,3	-6,6	-0,8	-4,8	
	BLIPS*	Count	0	11	0	0	14(a)	25
		Adjusted Residual	-3,2	7,7	-4,3	-0,5	**3,7**	
	APS**	Count	5(b)	0	79	0	0	84
		Adjusted Residual	-5,6	-3,3	13,1	-1,2	-6,9	
	GRD	Count	0	0	0	2	0	2
		Adjusted Residual	-0,9	-0,4	-1,2	14,6	-0,8	
	Psychotic	Count	1(c)	4(d)	5(e)	0	40	50
		Adjusted Residual	-4,5	0,3	-4,9	-0,8	10,1	
	Total	Count	57	15	84	2	54	212

APS, Attenuated Psychosis Group; BLIPS, Brief Limited Intermittent Psychotic Symptoms; CAARMS, Comprehensive Assessment of At Risk Mental States; GRD, Genetic Risk and Deterioration Syndrome; SIPS, Structured Interview for Prodromal Syndromes; UHR, Ultra High Risk; *, 4 BLIPS and GRD collapsed in BLIPS; **, 5 APS and GRD collapsed in APS; adjusted residuals lower than -3,29 or greater then 3,29 indicate significant differences at p<0.001 corrected for multiple comparison. In red significant off tangent residuals. Further qualitative analysis is appended below here:

(a) These BLPS subjects (on the CAARMS) presented with disorganizing or dangerous symptoms which qualify for psychosis on the SIPS (see eTable 4 below here for details)

(b). These subjects presented with APS symptoms (on the CAARMS) that were “better explained by another Axis-I or II disorder” (UHR-) on the SIPS

(c) This subject was psychotic on the CAARMS because of perceptual abnormalities only (severity 5 on P3) but not psychotic and with symptoms“better explained by another Axis-I or II disorder” (UHR-) on the SIPS

(d) These subjects were BLIPS lasting more than 7 days on the SIPS which qualify for psychosis on the CAARMS

(e) These subjects were APS (on the SIPS) only because of perceptual abnormalities (severity 5 on P3) which qualified for psychosis on the CAARMS

eTable 4. *Psychopathological features of 14 subjects diagnosed as BLIPS by the CAARMS and as Psychotic by the SIPS*

Clients ID	**CAARMS Outcome**	**SIPS Outcome**
1	*BLIPS*	*Psychotic*<br>Subject presenting with disorganizing hallucinatory symptoms (voices of an unknown girl) affecting her behaviour and familiar environment. Duration: 4 days. Not drug-induced.
2	*BLIPS*	*Psychotic*<br>Subject presenting with disorganizing persecutory ideation (idiosyncratic connections between personal events and beliefs that people were against him) seriously damaging his social relations and his job. Duration: 5 days. Not drug-induced.
3	*BLIPS*	*Psychotic*<br>Subject presenting with disorganizing hallucinatory symptoms and grandiose ideas (visions of angels and beliefs that he was a messenger of God), seriously damaging her work and social relations. Duration: 3-4 days. Not drug-induced.
4	*BLIPS*	*Psychotic*<br>Subject presenting with disorganizing behaviour and speech, seriously damaging his social relationships. Duration: 1 day. Cannabis related.
5	*BLIPS*	*Psychotic*<br>Subject presenting with seriously disorganizing religious beliefs and persecutory ideas (i.e. being chased by secret agencies), severely damaging her daily life and relations with her family. Duration: 5 days. Cannabis related.
6	*BLIPS*	*Psychotic*<br>Subject presenting with disorganizing and dangerous persecutory ideation. Aggressive towards objects (smashed a door to get out of his house and ran into the street due to belief of being chased by strangers). Duration: 4 days. Not drug-induced.
7	*BLIPS*	*Psychotic*<br>Subject presenting with disorganizing persecutory ideas (beliefs that his colleagues were plotting against him), severely damaging his behaviour and work relationships. Duration: 6-7 days. Not drug-induced.
8	*BLIPS*	*Psychotic*<br>Subject presenting with disorganizing mystical and persecutory ideation (beliefs that she was in contact with evil spirits), damaging her behaviour and consequently her personal dignity. Duration: 4-5 days. Not drug-induced.
9	*BLIPS*	*Psychotic*<br>Subject presenting with disorganizing persecutory ideas, seriously damaging his behaviour and his social and familiar relationship. Duration: 4 days. Not drug-induced.

10	*BLIPS*	*Psychotic*<br>Subject presenting with disorganizing somatic ideas and hallucinatory phenomena (beliefs parts of her body were not working properly), seriously damaging her personal dignity. Duration: 4-5 days. Not drug-induced.
11	*BLIPS*	*Psychotic*<br>Subject presenting with disorganizing referential and persecutory ideas (being watched by the neighbours and strangers in the street), seriously damaging his social interactions. Duration: 4 days. Not drug-induced.
12	*BLIPS*	*Psychotic*<br>Subject presenting with severe disorganizing and dangerous persecutory ideas. Aggressive and dangerous towards people and property (he punched one member of his family). Duration: 5-6 days. Not drug-induced.
13	*BLIPS*	*Psychotic*<br>Subject presenting with disorganizing persecutory ideation (strangers trying to give him threatening messages), seriously damaging his family relationships and his behaviour. Duration: 2-3 days. Not drug-induced.
14	*BLIPS*	*Psychotic*<br>Subject presenting with disorganizing persecutory ideas and disorder of thoughts, severely damaging her behaviour and her social skills. Duration: 3 days. Not drug-induced.

BLIPS: Brief Limited Intermittent Psychotic Symptoms; CAARMS, Comprehensive Assessment of At Risk Mental States; SIPS, Structured Interview for Prodromal Syndromes.

The notion of "seriously disorganized and dangerous" is introduced in the SIPS manual at the pages 14-15 with the concept of "urgency" ***[2]***. This is defined as follows:
*"Urgency is any positive psychotic symptom that is seriously disorganizing or dangerous no matter what the duration**[2]**".*

Further details are provided at page 31 with the comparative SIPS vs CAARMS table, and at page 50 with a clinical example***[2]***. The latter is described as follows:
"*An example of a 6 rating on perceptual abnormalities is a patient reporting that he hears the devil speaking to him and telling him to hurt himself. He believes the voice is real and he believes that he should act on the command.*
*This symptom meets criteria for being dangerous as well, and the patient would immediately meet criteria for current psychosis **[2]**"*

Overall the above cases were screened against the definition provided by the authors of the SIPS, as indicated in the main text: *""Dangerous' is taken to mean physically dangerous e.g. risk of death or serious physical injury, and 'disorganizing' means potentially psychosocially dangerous, e.g. risk of seriously damaging work relations, social relations, family relations, or personal dignity""*

eTable 5. Validation of CONVERT in the external independent sample of 93 subjects assessed with both CAARMS 12/2006 and SIPS 5.0 at the Seoul Youth Clinic.

**eTable 5A. SIPS to CAARMS with CONVERT**			**Gold standard CAARMS 12/2006 Seoul**		
			UHR+	UHR-	Total
**Test CAARMS 12/2006 CONVERT**	UHR+	Count	26	2	28
	UHR-	Count	5	60	65
	Total	Count	31	62	93

CAARMS, Comprehensive Assessment of At Risk Mental States; SIPS, Structured Interview for Psychosis Risk Syndromes; UHR, Ultra High Risk.

**eTable 5B. CAARMS to SIPS with CONVERT**			**Gold Standard SIPS 5.0 Seoul**		
			UHR+	UHR-	Total
**Test SIPS 5.0 CONVERT**	UHR+	Count	86	1	87
	UHR-	Count	0	6	6
	Total	Count	86	7	93

CAARMS, Comprehensive Assessment of At Risk Mental States; SIPS, Structured Interview for Psychosis-Risk Syndromes; UHR, Ultra High Risk.

REFERENCES TO SUPPLEMENTARY MATERIAL

[1] Yung, A. R.; Phillips, L. J.; Yuen, H. P.; McGorry, P. D., "*Comprehensive Assessment of at Risk Mental State.,"* Parkville Victoria, The PACE Clinic, ORYGEN Research Centre, University of Melbourne, Department of Psychiatry,2006.

[2] McGlashan, T. H.; Walsh, B.; Wood, S. J., "*The Psychosis-Risk Syndrome. Handbook for Diagnosis and Follow-up.,"* Oxford University Press: New York,2010.

[3] Fleiss, J., "The equivalence of weighted kappa and the intraclass correlation coefficient as measures of reliability," *Educational and Psychological Measurement,* vol. 33, no., pp.613-619, 1973.

[4] Albano, A. Equate: Observed-Score Linking and Equating. R package version 2.0-3. 2014. http://cran.r-project.org/web/packages/equate/index.html (accessed December 9th, 2014).

[5] Leucht, S., et al., "Linking the PANSS, BPRS, and CGI: clinical implications," *Neuropsychopharmacology : official publication of the American College of Neuropsychopharmacology,* vol. 31, no. 10, pp.2318-25, 2006.

[6] Leucht, S.; Rothe, P.; Davis, J. M.; Engel, R. R., "Equipercentile linking of the BPRS and the PANSS," *European neuropsychopharmacology : the journal of the European College of Neuropsychopharmacology,* vol. 23, no. 8, pp.956-9, 2013.

[7] Fusar-Poli, P., et al., "Disorder, not just a state of risk: meta-analysis of functioning and quality of life in subjects at high clinical risk for psychosis," *British Journal of Psychiatry,* vol., no., pp.in press, 2015.
